# Case Report: Cardiac Tamponade in Association With Cytokine Release Syndrome Following CAR-T Cell Therapy

**DOI:** 10.3389/fcvm.2022.848091

**Published:** 2022-03-21

**Authors:** Shohei Moriyama, Mitsuhiro Fukata, Taku Yokoyama, Shohei Ueno, Takuya Nunomura, Yasuo Mori, Koji Kato, Toshihiro Miyamoto, Koichi Akashi

**Affiliations:** ^1^Department of Hematology, Oncology and Cardiovascular Medicine, Kyushu University Hospital, Fukuoka, Japan; ^2^Department of Hematology, Hiroshima Red Cross Hospital & Atomic-bomb Survivors Hospital, Hiroshima, Japan; ^3^Department of Hematology, Faculty of Medicine, Institute of Medical Pharmaceutical and Health Sciences, Kanazawa University, Ishikawa, Japan

**Keywords:** cardiac tamponade, pericardial effusion, CAR-T, CRS, pericarditis

## Abstract

Chimeric antigen receptor T (CAR-T) cell therapy has been shown to have substantial efficacy against refractory hematopoietic malignancies. However, it frequently causes cytokine release syndrome (CRS) as a treatment-specific adverse event. Although cardiovascular events associated with CAR-T cell therapy have been increasingly reported recently, pericardial disease is a rare complication and its clinical course is not well characterized. Here, we report a case of acute pericardial effusion with cardiac tamponade after CAR-T cell therapy.

**Case Summary:**

A 59-year-old man with refractory diffuse large B-cell lymphoma underwent CAR-T cell therapy. Grade 2 CRS was observed on day 0; it progressed to grade 4 on day 7 and was accompanied by a fever over 39°C, hypoxia requiring intubation, hypotension requiring the use of a vasopressor agent, and supraventricular tachycardia. Although cardiac function was preserved, marked pericardial effusion with the collapse of the right heart was detected on echocardiography. Since pericardiocentesis was considered to have a high complication risk due to severe myelosuppression, medications for CRS were prioritized. Tocilizumab, an interleukin-6 inhibitor, and high-dose methylprednisolone (1 g/day for 3 days) were administered for the management of severe CRS. On day 8, the pericardial effusion decreased, and the hemodynamic status markedly stabilized. CRS did not exacerbate after the steroid dose was reduced. Further, lymphoma size reduced after the induction of CAR-T cell therapy, and tumor regrowth was not noted at 3 months after CAR-T cell infusion.

**Conclusion:**

Interleukin-6 pathway inhibitors and corticosteroid therapy should be considered in the context of CRS for significant pericardial effusion after CAR-T cell therapy in the acute phase.

## Introduction

Chimeric antigen receptor T (CAR-T) cell therapy is a novel immunotherapy for cancer treatment. Clinical trials of CAR-T cell therapy for refractory large B-cell lymphoma have shown a high response rate and long-term response (1, 2). However, CAR-T cells induce cytokine release syndrome (CRS), which is caused by a rapid increase in inflammatory cytokines released from activated immune cells and could be fatal (3). Cardiovascular complications such as heart failure, arrhythmia, and pericardial disease due to CAR-T cell therapy have been reported and shown to be strongly related to CRS. However, the clinical features and management of CRS have not yet been well defined (4, 5). Herein, we describe a case of acute pericardial effusion with cardiac tamponade associated with CRS after CAR-T cell therapy.

## Case Description

**Figure 1:**
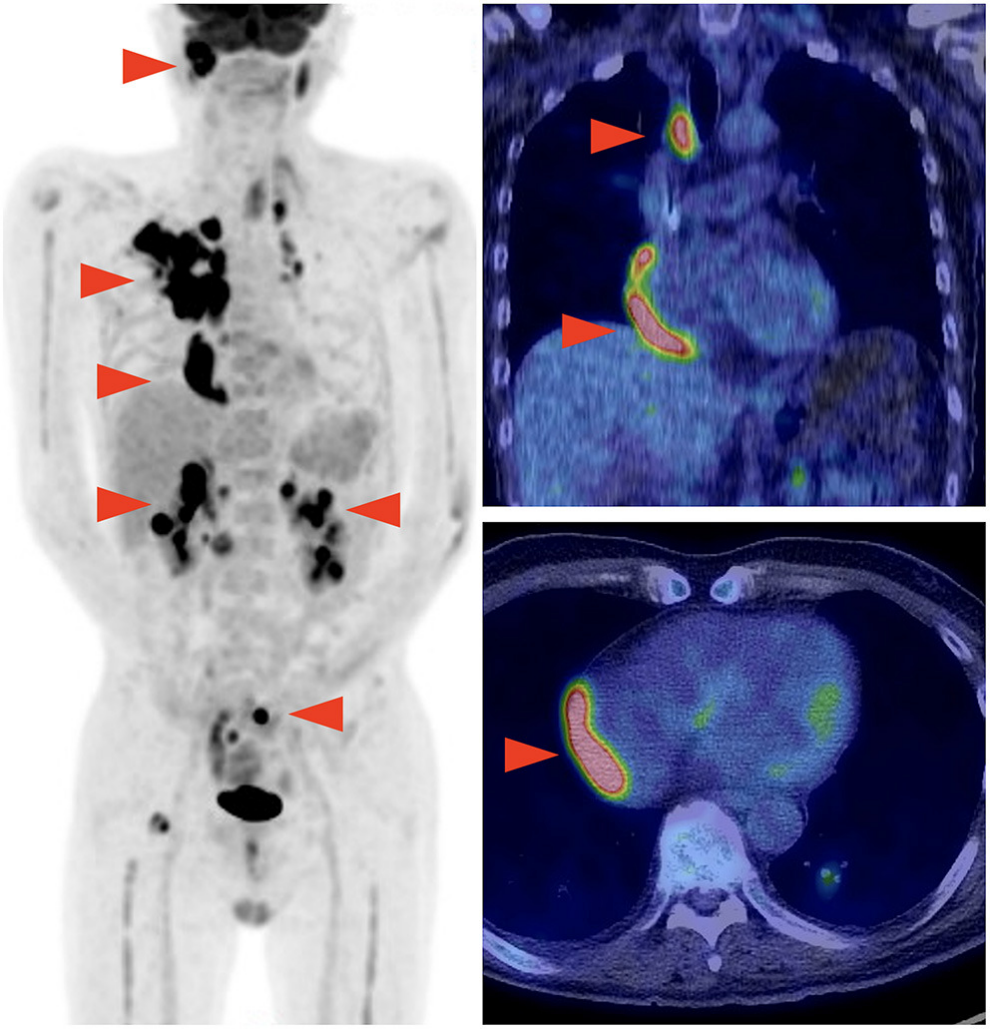
Positron emission tomography-computed tomography performed before chimeric antigen receptor T (CAR-T) cell therapy showing multiple lymphoma lesions including the site adjacent to the right pericardium.

**Figure 2:**
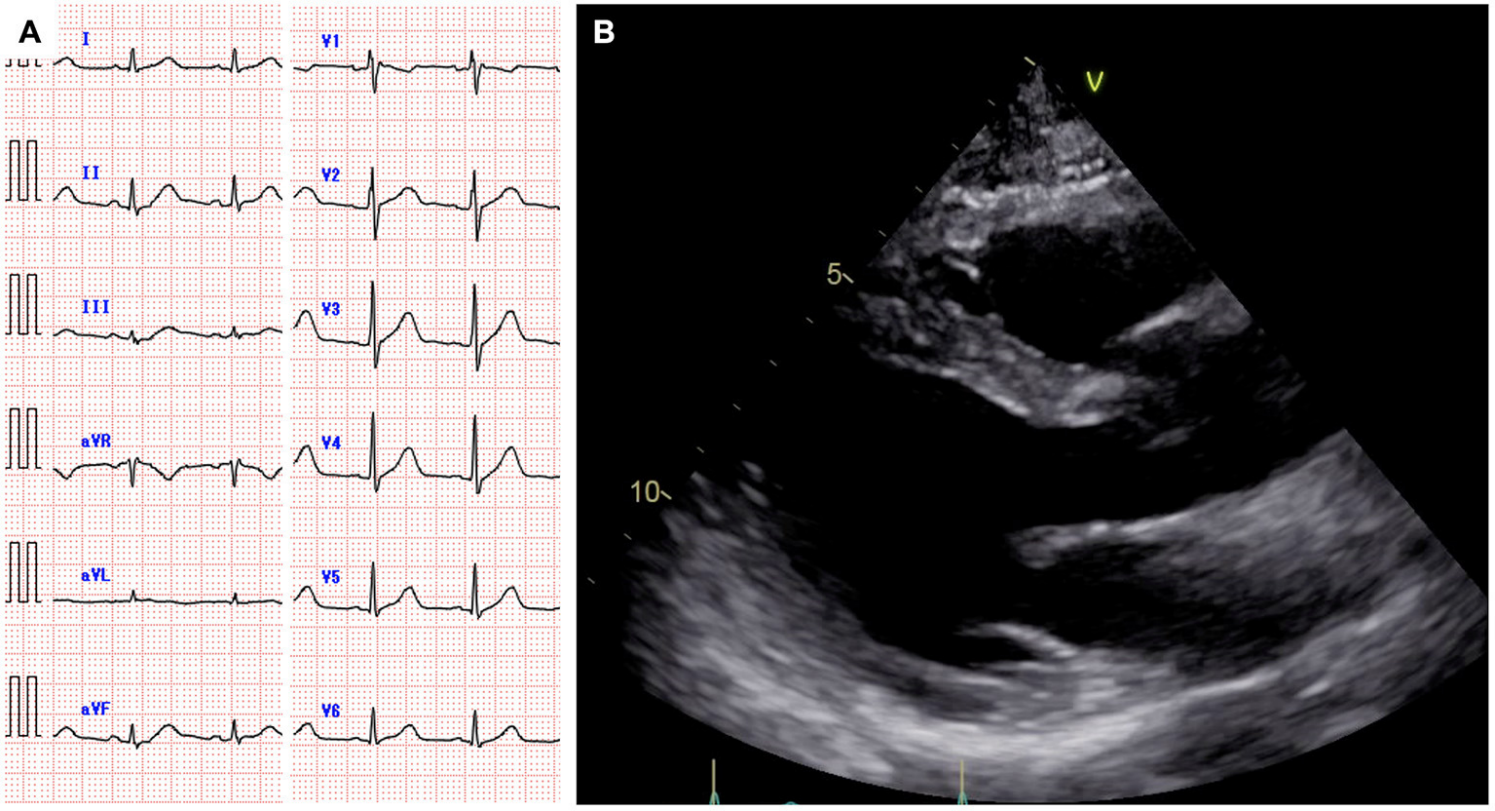
Electrocardiogram showing normal sinus rhythm **(A)**. Echocardiography showing normal cardiac function and no pericardial effusion **(B)**.

A 59-year-old man was diagnosed with diffuse large B-cell lymphoma (DLBCL), not otherwise specified. DLBCL relapsed after first-line chemotherapy with rituximab, cyclophosphamide, doxorubicin, vincristine, and prednisolone. Despite the multiple salvage chemotherapies and local radiation therapy, it aggravated and systemic multiple lymphoma lesions developed. Part of the lesions in the mediastinum was adjacent to the right side of the pericardium (Figure 1). The patient was referred to our hospital for anti-CD19 CAR-T cell therapy with axicabtagene ciloleucel as part of a phase 2 clinical trial (JapicCTI-183914; https://rctportal.niph.go.jp/en/detail?trial_id=JapicCTI-183914) (6). He had a history of a duodenal ulcer but not cardiovascular disease. The cumulative anthracycline dose administered was 440 mg/m^2^. The radiation therapy was performed for supraclavicular lesion with a dose of 46Gy 8 months ago and sacral lesion with a dose of 30Gy 3 months ago, and both site did not include the heart. An electrocardiogram obtained before the administration of CAR-T therapy showed normal sinus rhythm (Figure 2A), and echocardiography showed normal left ventricular systolic and diastolic functions with no pericardial or pleural effusion (Figure 2B, Supplementary Movie 1).

**Figure 3:**
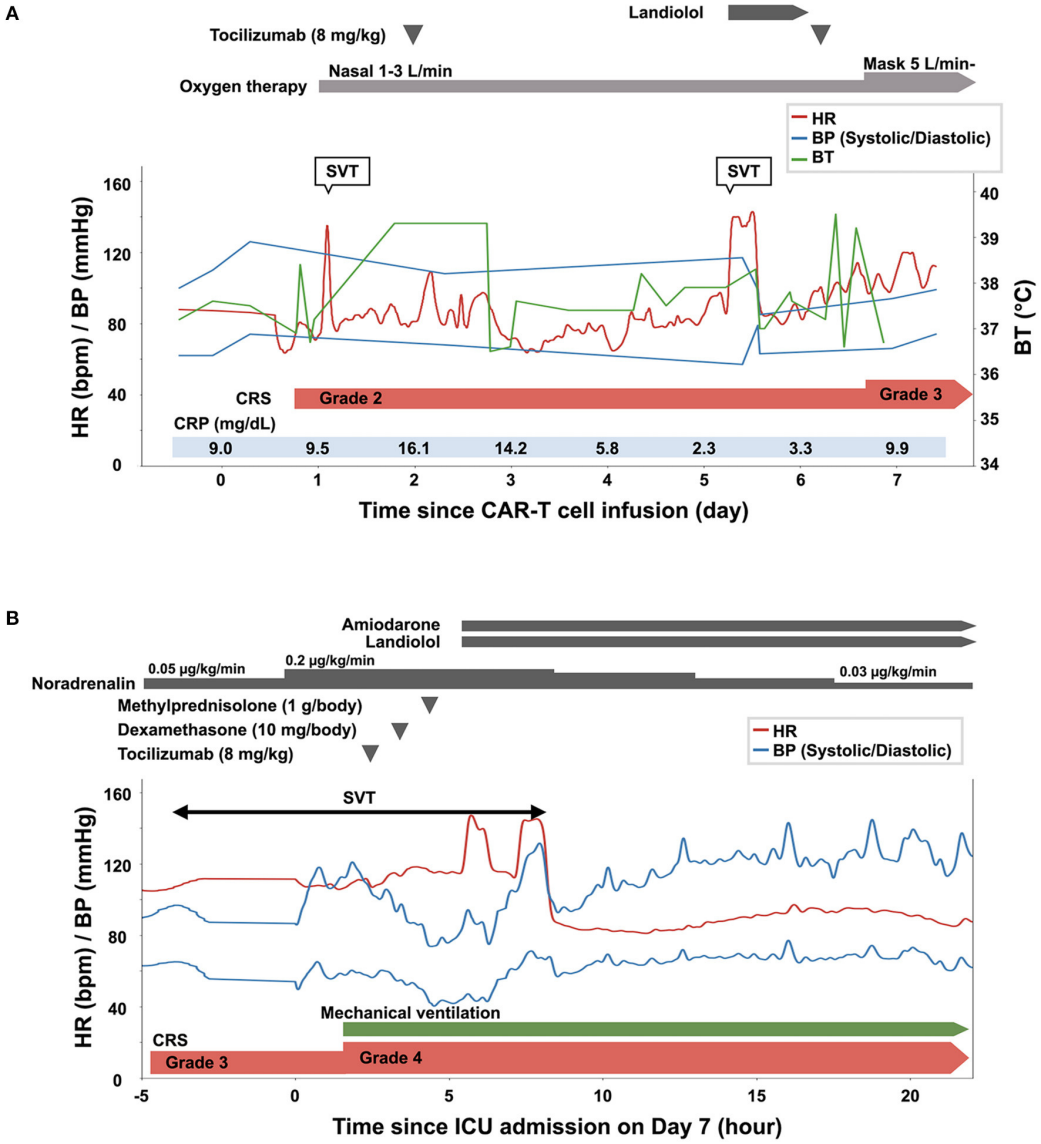
Clinical course of the patient from the day of the infusion of chimeric antigen receptor T (CAR-T) cells (day 0) until day 6 **(A)**, and on day 7 **(B)**. BP, blood pressure; BT, body temperature; CRP, C-reactive protein; CRS, cytokine release syndrome; ICU, intensive care unit; SVT, supraventricular tachycardia.

Following conditioning chemotherapy with cyclophosphamide and fludarabine, CAR-T cells (2.0 × 10^6^ cells/kg) were administered. Empiric administration of antibiotics was initiated on day 0. The patient developed fever (≥38°C) on the day of CAR-T cell infusion (day 0) and hypoxia requiring oxygen on day 1. He was diagnosed with grade 2 CRS (7), and tocilizumab (8 mg/kg) was administered for the same on day 2 (Figure 3A). Fever due to bacterial infection was ruled out based on negative blood culture results. He developed supraventricular tachycardia on day 5, and landiolol was administered. Computed tomography on day 5 showed marked pleural effusion and slight pericardial effusion (Supplementary Figure 1). Because grade 2 CRS persisted, tocilizumab (8 mg/kg) was readministered on day 6. However, exacerbation of hypoxia and severe hypotension with tachycardia was observed on day 7 (CRS grade 3), and the patient was transferred to the intensive care unit.

**Figure 4:**
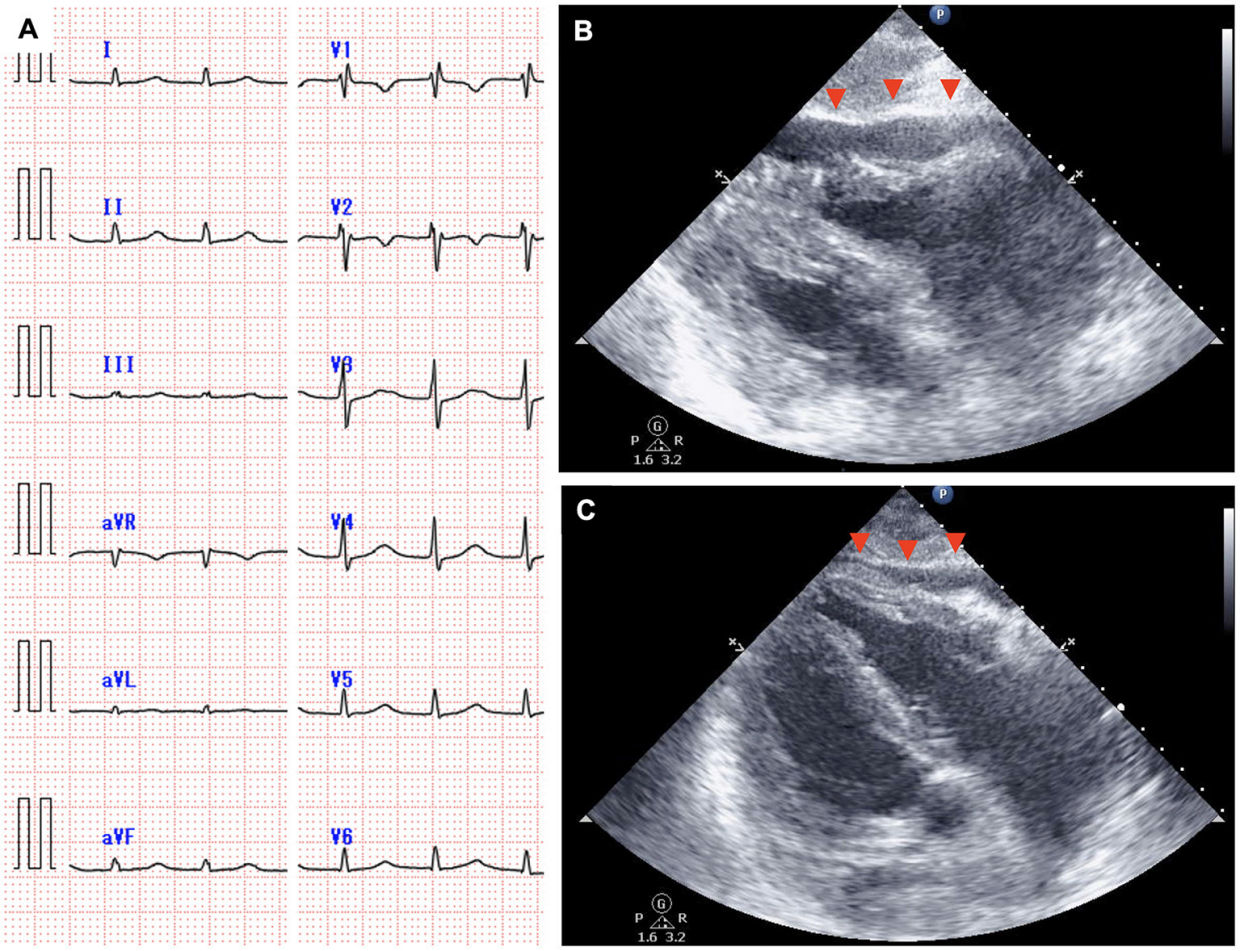
An electrocardiogram obtained on day 7 showing narrow QRS tachycardia with the absence of the P wave **(A)**, and echocardiography showing rapidly accumulated pericardial effusion with the collapse of the right heart chamber [**(B)**, arrowhead]. Pericardial effusion decreased and the collapse of the right heart chamber resolved on day 8 [**(C)**, arrowhead].

The time course of the vital signs and medications on day 7 is shown in Figure 3B. His blood pressure was 67/40 mmHg with noradrenalin (0.2 μg/kg/min), heart rate was 115 beats per minute, body temperature was 38.9°C, and percutaneous oxygen saturation was 86% with oxygen (10 L/min) administered through a face mask with a reservoir bag. The patient was intubated soon after admission to the intensive care unit because of his deteriorating respiratory condition (CRS grade 4). Jugular venous distension and pretibial pitting edema were observed. Furthermore, heart sounds were distant. Pericardial friction rubs were not evident. Serum C-reactive protein (CRP) was elevated to 9.9 mg/dL (Figure 3A, Supplementary Figure 2). Serum troponin T (0.038 ng/mL; normal range: ≤ 0.014 ng/mL) and brain natriuretic peptide (323.3 pg/mL; normal range: ≤ 18.4 pg/mL) levels were also elevated. An electrocardiogram revealed supraventricular tachycardia (Figure 4A). Echocardiography revealed preserved bilateral ventricular systolic functions but marked pericardial effusion (maximum echo-free space: 15 mm at end-diastole). The right heart chamber collapsed in the early diastolic phase, and the inferior vena cava was distended with a reduced respiratory diameter change (Figure 4B, Supplementary Movies 2, 3). Based on these findings, the patient was diagnosed with severe CRS and cardiac tamponade.

Although pericardiocentesis would have been effective for hemodynamic stabilization, immediate pericardiocentesis was deferred, because of the high complication risk associated with the procedure owing to severe myelosuppression (white blood cell count, 590/μL; platelet count 31,000/μL). The negative blood culture results and the rapid increase in pericardial effusion despite the administration of antibiotics suggested that the cause of pericardial effusion was not a bacterial infection. Therefore, immunosuppressive medications were prioritized for CRS management. Since blood pressure remained low (<80/ <50 mmHg) after the administration of tocilizumab followed by dexamethasone (10 mg/body), intravenous high-dose methylprednisolone (1 g/body) was administered. The patient's blood pressure increased 2 h after the administration of methylprednisolone. Amiodarone and landiolol were initiated for the treatment of recurrent supraventricular tachycardia. On the next day (day 8), pericardial effusion decreased, and echocardiography showed attenuation of the degree of right heart chamber collapse and recovery of respiratory diameter change of inferior vena cava (Figure 4C, Supplementary Movies 4, 5). The patient's blood pressure and heart rate improved further and stabilized. Serum CRP consistently decreased after the administration of high-dose methylprednisolone, and the steroids dose was gradually reduced until discontinuation on day 29 (Supplementary Figure 2). Echocardiography after the discontinuation of steroid therapy showed no pericardial effusion and normal biventricular systolic function. Supraventricular tachycardia was occasionally observed up to 1 month after CAR-T cell infusion. Computed tomography on day 91 showed a partial response of the primary disease to CAR-T cell therapy. The patient was eventually discharged on day 112. There was no cardiovascular adverse event after discharge. At 6 months after the CAR-T cell therapy, the lymphoma aggravated rapidly and the treatment for this patient was transferred to best supportive care.

## Discussion

Anti-CD19 CAR-T cell therapy has been approved for the management of adult non-Hodgkin lymphoma and leukemia in young adults or children globally and is expected to be expanded to other malignancies in the future. Refractory DLBCL was reported to have an abysmal prognosis, with a median overall survival of 6.3 months and a response rate of 26% even after chemotherapy and hematopoietic stem cell transplantation before the CAR-T cell therapy era (8). Anti-CD19 CAR-T cell therapy has markedly improved the prognosis of patients with refractory large B-cell lymphoma, with a response rate increase to 52–82% and longer overall survival (1, 2, 9). However, this novel therapy frequently causes CRS by over-activation of the immune system and subsequent elevation of cytokines such as interferon-gamma, tumor necrosis factor-alpha, and interleukin (IL)-6 (10). In recent years, there has been an increase in the number of reports on cardiovascular events after CAR-T cell therapy with or without overlapping CRS (4, 5). To our knowledge, this is the first report of a case of cardiac tamponade associated with CRS after CAR-T cell therapy.

Raza et al. reported that the rate of cardiotoxicity after CAR-T cell therapy in adult patients with lymphoma or multiple myeloma was 12% (11). In their study, when analyzing limited patients who developed grade 2-4 CRS, cardiovascular events were found to have occurred in 31% of the patients, whereas no cardiovascular events were observed in patients without or grade 1 CRS (11). Lefebvre et al. reported that major adverse cardiovascular events occurred in 17% of patients at 30 days, 19% at 6 months, and 21% at 12 months. The time from CAR-T cell infusion to the onset of cardiovascular events is similar to that of CRS which occurring in most cases within 30 days of CAR-T cell infusion (12). The relatively high incidence of cardiovascular events in cases with high-grade CRS and the simultaneous development of CRS and cardiovascular events suggest a causal relationship between CRS and cardiovascular toxicity in the early period following CAR-T cell infusion. In our case, fever was observed immediately after CAR-T cell infusion, followed by refractory hypotension and hypoxia in a week. This clinical course is consistent with the typical manifestation of high-grade CRS after CAR-T cell therapy in a previous report (13). Additionally, in this case, the development of CRS preceded pericardial effusion, and cardiac tamponade was observed with the exacerbation of CRS. CRP, a surrogate marker for IL-6 bioactivity with time lag by 1 to 2 days, peaked on the next day of the development of cardiac tamponade (7, 14). Taken together, the developmental course of pericardial effusion and its evident response to steroid therapy suggested a relationship between CRS and the development of cardiac tamponade.

Pericarditis or pericardial effusion after CAR-T cell therapy is rare, with a total incidence rate of 0.4%, and two-thirds of patients with pericardial disease have concurrent CRS (5). The underlying mechanism of pericardial disease after CAR-T cell therapy has not been well clarified. Cytokine-induced fluid retention and/or systemic high vascular permeability is a possible cause of rapid accumulation of pericardial fluid (15, 16). The clinical features of severe CRS resemble those of capillary leak syndrome (4, 5, 15, 16). Pericardial effusion is reported to occur in 11% of patients with idiopathic capillary leak syndrome and can be life-threatening (17, 18). Since the levels of inflammatory cytokines such as IL-6 and vascular endothelial growth factor significantly increased in the serum of patients with severe capillary leak syndrome, this cytokine-related capillary leak may induce the accumulation of pericardial fluid (19, 20). Hemodynamic deterioration in this case was accompanied by the dilatation of inferior vena cava, which is quite different from hypovolemic state in case of capillary leak syndrome (17). This clinical feature suggests the cause of pericardial effusion in this case includes a different mechanism other than capillary leakage. The difference in mediators between idiopathic capillary leak syndrome and CAR-T cell-induced CRS has not been fully elucidated. Accumulation of evidence from studies on the cytokine profiles and their signaling pathways in cases of CAR-T cell-induced CRS would help understand the mechanism of organ damage by CRS due to CAR-T cell therapy. An additional possible cause is locally accelerated pericardial inflammation. In this case, CAR-T cell immunoreaction to lymphoma lesion adjacent to the pericardium might have accelerated the accumulation of pericardial fluid. In addition, the highest engineered T-cell concentration was detected in the pericardium or the myocardium, and the levels of some cytokines in the pericardial fluid were higher than the corresponding levels in blood in a case of cardiogenic shock after T-cell receptor-engineered T-cell immunotherapy (21). Direct T-cell toxicity in the pericardium or the myocardium has not been demonstrated in anti-CD19 CAR-T cell therapy to date; however, it needs to be elucidated in the future (4).

IL-6 plays an important role in the pathogenesis of CRS, and tocilizumab, an IL-6 receptor antagonist, has been approved as first-line therapy for CRS (22, 23). For severe CRS with a poor response to tocilizumab, additional administration of corticosteroids is recommended (23). In previous reports, more than 50% of patients with CRS showed amelioration after tocilizumab administration, and no tocilizumab-related adverse reactions were detected (1, 2). Furthermore, corticosteroid therapy did not suppress the response to CAR-T cell therapy clinically; however, it decreased the number of CAR-T cells in the serum and bone marrow (1, 14, 24). Therefore, its use may have to be limited to life-threatening conditions associated with severe CRS. Consideration of infection before and after the administration of tocilizumab or corticosteroids is also important because of the immunocompromised state of the patients (23). In this case, given the negative findings of bacterial infection and the considerable risk of pericardiocentesis due to severe myelosuppression, the administration of high-dose corticosteroids in addition to tocilizumab took precedence over pericardiocentesis, and the patient's hemodynamic condition drastically improved. Nevertheless, for all cases of cardiac tamponade, the best precautions to perform prompt pericardiocentesis should be taken in case of hemodynamic deterioration. Pericardiocentesis should be considered even if the patient has severe thrombocytopenia if the response to high-dose steroid therapy in addition to tocilizumab is unfavorable (25).

## Conclusion

We described the case of a 59-year-old man with refractory DLBCL who was treated with anti-CD19 CAR-T cell therapy and developed severe CRS and cardiac tamponade. Appropriate immunosuppressive therapy for CRS reduces pericardial effusion without the need for pericardiocentesis. When hemodynamic instability occurs in the acute phase after CAR-T cell therapy, CRS-related cardiovascular complications including cardiac tamponade as well as severe CRS need to be considered. In cases in which pericardiocentesis poses a considerable risk, urgent strengthening of the treatment for CRS is worth considering.

## Data Availability Statement

The original contributions presented in the study are included in the article/Supplementary Material, further inquiries can be directed to the corresponding author.

## Ethics Statement

Written informed consent was obtained from the patient for the publication of any potentially identifiable images or data included in this article.

## Author Contributions

SM drafted the manuscript and all authors contributed significantly to the manuscript. All authors reviewed the draft manuscript and provided critique and feedback on the manuscript. All authors read and approved the final version of the manuscript.

## Funding

This work was supported by JSPS KAKENHI Grant Number JP17K11577.

## Conflict of Interest

KK received research funding from Chugai Pharmaceutical, Takeda Pharmaceutical, Kyowa Kirin, AbbVie, Novartis, Eisai, Janssen, Celgene, Ono Pharmaceutical, Daiichi Sankyo, and honorarium from Chugai Pharmaceutical, Takeda Pharmaceutical, Kyowa Kirin, Novartis. KA received research funding from Chugai Pharmaceutical, Takeda Pharmaceutical, Kyowa Kirin, AbbVie, Eisai, Ono Pharmaceutical, Asahi Kasei, Shionogi, Sumitomo Dainippon Pharma, Otsuka Pharmaceutical, Taiho Pharmaceutical, Astellas Pharma, and honorarium from AbbVie, Eisai. The remaining authors declare that the research was conducted in the absence of any commercial or financial relationships that could be construed as a potential conflict of interest.

## Publisher's Note

All claims expressed in this article are solely those of the authors and do not necessarily represent those of their affiliated organizations, or those of the publisher, the editors and the reviewers. Any product that may be evaluated in this article, or claim that may be made by its manufacturer, is not guaranteed or endorsed by the publisher.

## Supplementary Material

Supplemental Figure 1 Computed tomography performed on day 5 showing bilateral pleural effusion and slight pericardial effusion.

Supplemental Figure 2 Serum C-reactive protein (CRP) levels from the day of the infusion of chimeric antigen receptor T (CAR-T) cells until day 14. CRS, cytokine release syndrome.

## Abbreviations

CAR-T: Chimeric antigen receptor
CRS: Cytokine release syndrome
DLBCL: Diffuse large B-cell lymphoma
IL-6: Interleukin-6.
