# Supplementary figures and images for: Case Report: Cardiac Tamponade in Association With Cytokine Release Syndrome Following CAR-T Cell Therapy
Source: Front Cardiovasc Med. 2022 Mar 21;9:848091. doi: 10.3389/fcvm.2022.848091 (PMC8977736; doi:10.3389/fcvm.2022.848091)

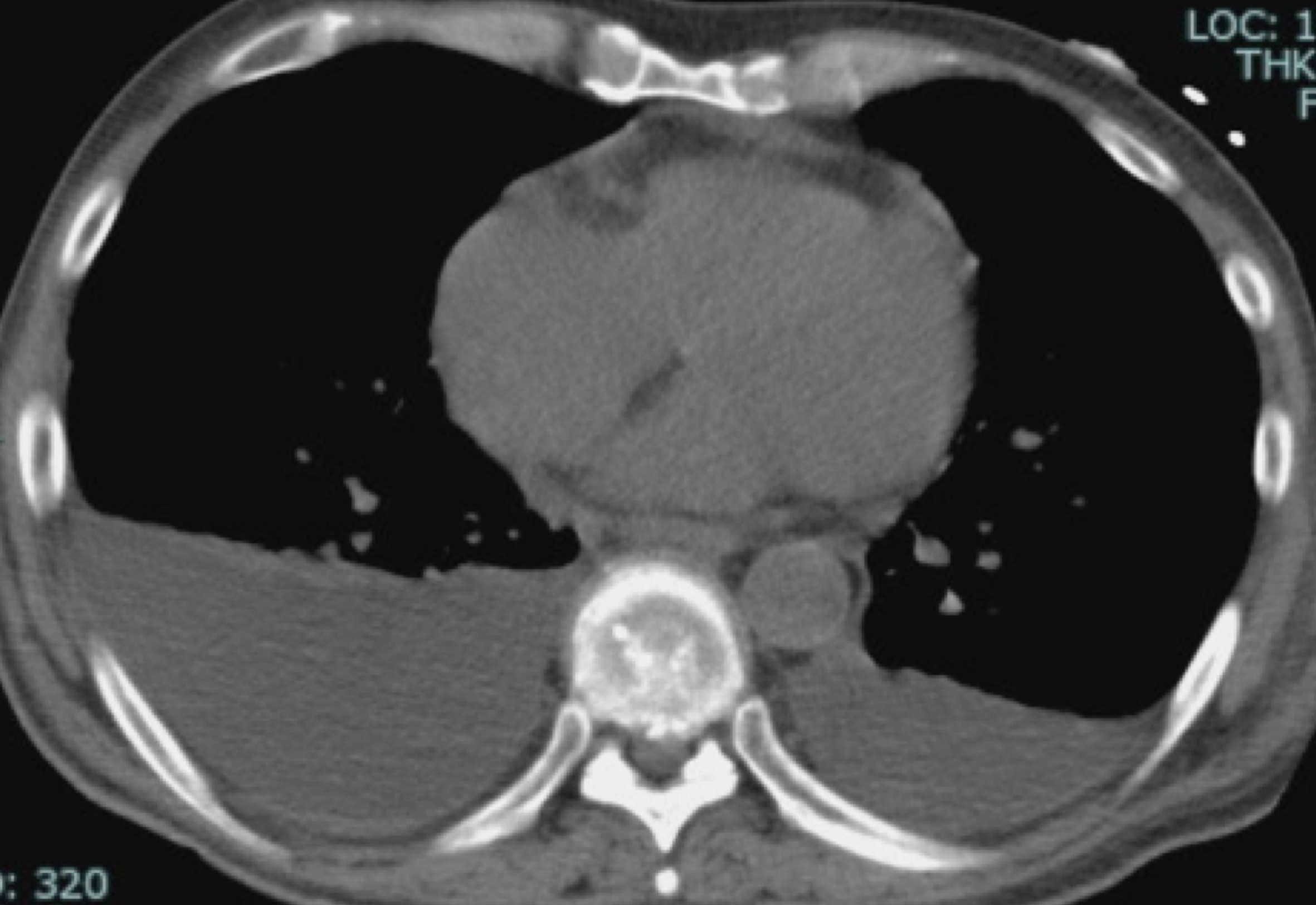

Supplement: Supplemental Figure 1 — Computed tomography performed on day 5 showing bilateral pleural effusion and slight pericardial effusion. [file Image_1.TIFF]

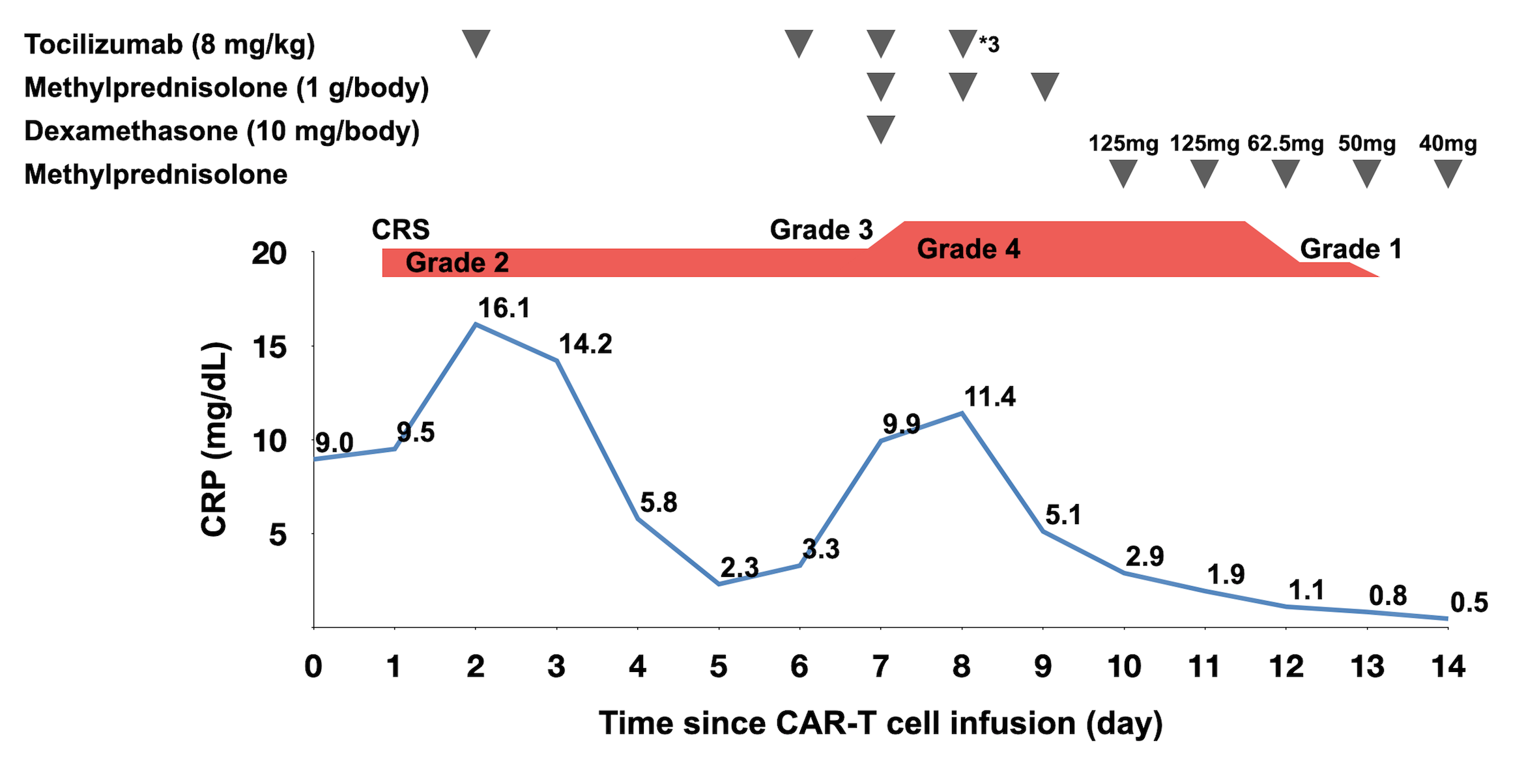

Supplement: Supplemental Figure 2 — Serum C-reactive protein (CRP) levels from the day of the infusion of chimeric antigen receptor T (CAR-T) cells until day 14. CRS, cytokine release syndrome. [file Image_2.TIFF]
